# Corrugated-Diaphragm Based Fiber Laser Hydrophone with Sub-100 μPa/Hz^1/2^ Resolution

**DOI:** 10.3390/s17061219

**Published:** 2017-05-26

**Authors:** Wen-Zhao Yang, Long Jin, Yi-Zhi Liang, Jun Ma, Bai-Ou Guan

**Affiliations:** Guangdong Provincial Key Laboratory of Optical Fiber Sensing and Communications, Institute of Photonics Technology, Jinan University, Guangzhou 510632, China; yangwenzhao@stu2014.jnu.edu.cn (W.-Z.Y.); liangyizhi@stu2014.jnu.edu.cn (Y.-Z.L.); jun.ma@jnu.edu.cn (J.M.); tguanbo@jnu.edu.cn (B.-O.G.)

**Keywords:** fiber laser sensors, acoustic sensors, fiber optic hydrophones, fiber Bragg grating

## Abstract

In this work, a beat-frequency encoded fiber laser hydrophone is developed for high-resolution acoustic detection by using an elastic corrugated diaphragm. The diaphragm is center-supported by the fiber. Incident acoustic waves deform the diaphragm and induce a concentrated lateral load on the laser cavity. The acoustically induced perturbation changes local optical phases and frequency-modulates the radio-frequency beat signal between two orthogonal lasing modes of the cavity. Theoretical analysis reveals that a higher corrugation-depth/thickness ratio or larger diaphragm area can provide higher transduction efficiency. The experimentally achieved average sensitivity in beat-frequency variation is 185.7 kHz/Pa over a bandwidth of 1 kHz. The detection capability can be enhanced by shortening the cavity length to enhance the signal-to-noise ratio. The minimum detectable acoustic pressure reaches 74 µPa/Hz^1/2^ at 1 kHz, which is comparable to the zeroth order sea noise.

## 1. Introduction

Fiber optic hydrophones have been developed to detect underwater acoustic waves for engineering and defense applications. Compared to their piezoelectric counterparts, fiber optic hydrophones have exhibited high sensitivity and immunity to electromagnetic interference. Their multiplexing capability has been improved by using fiber Bragg gratings and fiber lasers as sensing elements [1,2,3,4]. A number of sensor configurations have been demonstrated and optimized for better detection capability. The implementation of interferometric fiber optic hydrophones relies on the translation of acoustic pressure into a change in optical phase difference between the sensing and reference arms [4]. Recently, the responsivity has been greatly enhanced by using a hollow-core photonic bandgap fiber with a high air-filling ratio in transverse geometry as a sensing fiber [5]. In addition, fiber-tip Fabry-Perot cavities based on deformable diaphragms can be utilized as photonic hydrophones. The diaphragms can be made of metal nanolayers [6,7], photonic crystals [8,9], and even mono- or multi-layer graphenes [10]. The diaphragms deform and thus change the round-trip phase in response to acoustic waves. The noise-equivalent-acoustic pressure (NEAP) can reach μPa/Hz^1/2^ level, taking advantage of the highly deformable nature of the diaphragms. A fiber laser has been exploited as a hydrophone by measuring its lasing frequency variation as a result of acoustically induced longitudinal strain [11]. The frequency change can be demodulated with an imbalanced interferometer with high resolution. As a result, the pressure sensitivity at 1 kHz is typically 58.0 dB re μPa/Hz^1/2^, corresponding to a minimum detectable acoustic pressure of 800 μPa [12]. Further sensitivity enhancement has been achieved by using a hollow rugby-ball-like transducer [13].

Different from the single-frequency ones, a fiber laser sensor can also work in a beat-frequency encoding manner, that is, the *x-* and *y-* polarized lasing modes yield a beat signal whose frequency can shift in response to external perturbations [14,15,16]. This manner reserves multiplexing capability and the sensors can be cascaded to form an array [17]. The radio-frequency (RF) beat signal can be demodulated with methods that have been used in radio and microwave systems. This drift of carrier signal does not affect the extraction of the fast-varying signal and, therefore, the sensor offers inherent immunity to environmental perturbations. The beat-frequency encoded fiber laser has been used for underwater acoustic detection, as described in [14], but its detection capability was not fully exploited.

In this work, we successfully pushed the detection limit to sub 100 μPa/Hz^1/2^ level. A corrugated diaphragm is used to concentrate uniform acoustic pressure to a point load on the laser cavity and induces optical response in beat-frequency variation. Experiment results show that the average acoustic sensitivity is 185.7 kHz/Pa with a working bandwidth of about 1 kHz. Sensors with shorter laser cavities can present better detection capability to a point load, due to a higher sensitivity-to-noise ratio. As a result, the detection limit at 1 kHz reaches 74 µPa/Hz^1/2^, which is comparable to the zeroth order sea noise.

## 2. Working Principle

The sensing element is a dual-polarization-mode fiber laser, fabricated by photoinscribing two wavelength-matched Bragg gratings in a single mode Er-doped optical fiber (M-12, Fibercore, Southampton, UK) to form a Fabry-Perot cavity. The fiber has a cutoff wavelength at around 935 nm and an absorption of 12 dB/m at 980 nm. The reflective wavelength of each grating is located at 1530 nm. The grating lengths are 3.5 and 4 mm, respectively. The coupling strength of each grating is higher than 25 dB (99.7% in reflectivity) to provide strong optical feedbacks for lasing oscillation. The output power of the laser is typically 100 μW with a pump power of 200 mW. The grating separation *L_s_* is less than 8 mm in our experiment to obtain single-longitudinal mode output. With the injection of pump light at 980 nm, such a cavity intrinsically emits two orthogonal polarization lasing modes at frequencies *ν_x_* and *ν_y_*, respectively. The beat frequency can be expressed by $\Delta \nu =\left|\nu_{x}-\nu_{y}\right|=cB/n_{0}\;\lambda$, where *B* represents the fiber birefringence, *c* is the speed of light in vacuum, *n_0_* is the average effective modal index, and *λ* denotes the lasing wavelength. Acoustic pressure is translated into birefringence change, which induces a beat-frequency variation. The beat signal is mixed with two local RF signals with 90 degree phase offset. This down-conversion treatment generates I/Q data which depicts the phase of the beat signal [18]. The frequency variation is obtained by taking a derivation of the phase change over time.

**Figure 1:**
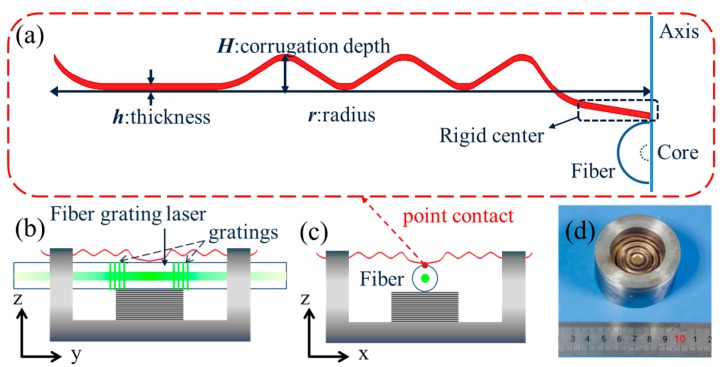
(**a**) Schematic of the radial profile of the elastic corrugated diaphragm and the sensing element, i.e., the fiber grating laser. The laser is placed beneath and in contact with the rigid center of the diaphragm. The diaphragm translates applied acoustic pressure into a lateral load on the laser via point contact; (**b**) Sectional view (y-z plane) of the packaged sensor; (**c**) Sectional view (x-z plane) of the packaged sensor; (**d**) Photograph of the packaged sensor.

Figure 1 shows the schematic of the present sensor. An elastic corrugated diaphragm is selected to effectively translate the acoustic wave into a lateral load on the fiber. This kind of diaphragm has been widely used as a basic pressure sensitive element in engineering applications. The diaphragm has a circularly symmetrical geometry with three corrugation periods along the radial direction. As shows in Figure 1a, the diaphragm has a point contact with the optical fiber at the rigid center. The diaphragm is made of beryllium bronze. It has a radius *r* = 19 mm, a thickness *h* = 0.2 mm, and a corrugation depth of *H* = 2 mm. Figure 1b,c demonstrates side views of the sensor package. The fiber laser is fixed in a cylindrical base and a metal block is used to support the fiber to give a pre-charge for better stability. Figure 1d shows the photograph of the sensor package.

**Figure 2:**
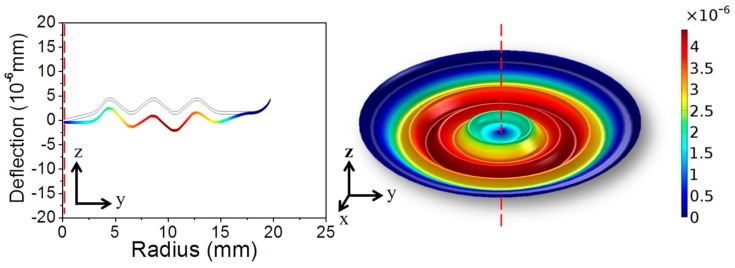
FEM calculated deformations of an edge-clamped/center-supported corrugated diaphragm in response to 10 Pa static pressure.

Acoustic pressure applied at the upper surface tends to deform the diaphragm and induce a vertical deflection at the rigid center (See Appendix A). In contrast, a concentrated force −F normally applied at the rigid center can induce a deflection *d*_0_ [19]

(1) $$d_{0}=-F\frac{r^{2}}{A^{\prime }\pi Eh^{3}}$$

where $A^{\prime }={\left(1+q\right)}^{2}/3\left(1-\mu^{2}/q^{2}\right)$. Figure 2 shows the numerical result of the deformation of a center-supported diaphragm. In the present hydrophone, the center-supported/edge clamped diaphragm has a zero deflection as a result of the balance between the applied pressure and the reacting force from the fiber. Combining Equations (1) and (A2), the balance between the acoustic pressure and the point load given by the fiber can be expressed as

(2) $$F=-P\cdot A_{e}$$

where $A_{e}=\Gamma \pi r^{2}$ denotes the effective area of corrugated diaphragm, Γ=(1+q)/2(3+q) is defined as a transduction factor. Equation (2) suggests that the uniform pressure *P* is concentrated to a point load *F* onto the laser cavity. The transduction efficiency scales with the area of the diaphragm *πr*^2^ as well as the factor Γ which is determined by the shape of the corrugation.

The optical response in beat-frequency change induced by the point load *F* can be written as [20]

(3) $$\delta \left(\Delta \nu \right)=\frac{c\gamma \eta }{n_{0}\lambda }\cdot F$$

where γ denotes the birefringence change rate with unit linear force density (N/m), which is mainly determined by the elastic properties and diameter of the glass fiber [21]. The product $c\gamma /n_{0}\lambda$ has an amplitude of around 10 GHz/(N/mm). The factor *η* is the normalized local intensity, which can be expressed by $\eta =\underset{L_{c}\to 0}{\lim}\frac{\int_{-L_{c}/2}^{+L_{c}/2}{\left|e(z)\right|}^{2}dz}{L_{c}}$, where |*e*(*z*)|^2^ represents the laser mode profile, *L*_c_ is the contact length between the fiber and the diaphragm. Here we assume that the laser mode is totally confined by the gratings and has a nearly uniform intensity over the blank region between the gratings. This approximation is reasonable with *L*_s_ > 2 mm, considering the strong grating back coupling and the relatively low fiber gain [20]. This approximation leads to $\eta =1/L_{s}$ and the optical response in beat-frequency change induced by the point load *F* can be written as [20],

(4) $$\delta \left(\Delta v\right)≐\frac{c\gamma }{n_{0}\lambda }\cdot \frac{F}{L_{s}}$$

*L_s_*, once again, denotes the grating separation that represents the length between two gratings.

**Figure 3:**
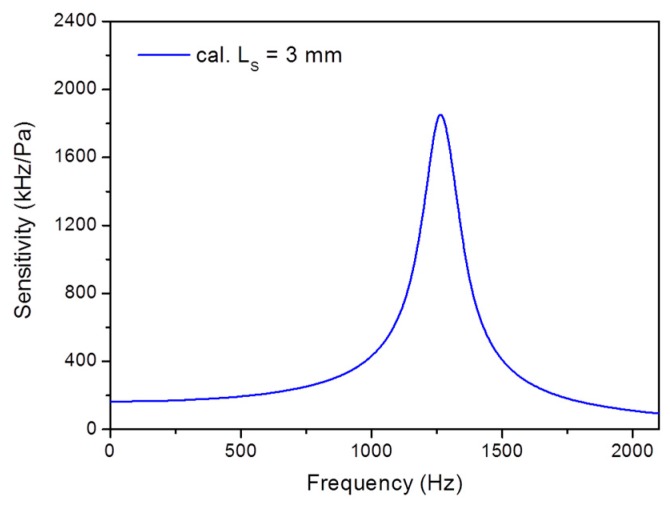
Calculated frequency response of fiber laser hydrophone with *L_s_* = 3 mm and *ξ* = 0.04.

Figure 3 shows the calculated frequency response, based on Equations (2), (4) and (A4). The first-order resonant frequency is *f_00_* = 1269 Hz. The calculated static pressure sensitivity is 164.3 kHz/Pa for *L_s_* = 3 mm. In the calculation, the damping factor is set as *ξ* = 0.04, which is a result of the damping effect of the vibrating diaphragm in water.

## 3. Experimental Setup

**Figure 4:**
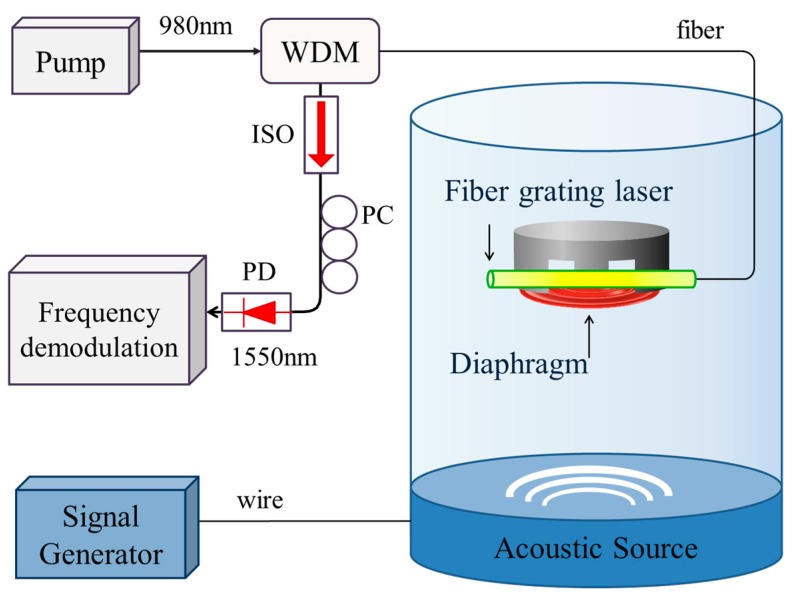
Schematic of acoustic detection with the fiber grating laser hydrophone. WDM: Wavelength-division multiplexer; ISO: Optical isolator; PD: Photodetector; PC: Polarization controller.

Figure 4 shows the experimental setup of acoustic detection with a corrugated-diaphragm based fiber laser hydrophone. The fiber laser is pumped with a 980 nm laser diode via a wavelength-division multiplexer (WDM). The inner space of the transducer is filled with water for pressure balance. The water tank is a cylindrical one with a height of 0.4 m and a radius of 0.16 m. The water depth is 0.36 m. A waterproof speaker (UWS-045, KHZ Corporation, Zhongshan, China) is placed at the bottom of a water tank as an acoustic source. The distance between the acoustic source and the transducer is 0.2 m in our experiment. The sensor is in the far field of the acoustic source. A stable stationary wave field can be formed by the upward acoustic wave emitted by the source and the reflected wave by the surface of water. The amplitude and frequency of the acoustic wave can be adjusted by a digital signal generator. The acoustic frequency scans from 110 to 2010 Hz with a step of 50 Hz. A commercial hydrophone (8104, Brüel&Kjær, Copenhagen, Denmark) is used for calibration.

## 4. Results and Discussion

**Figure 5:**
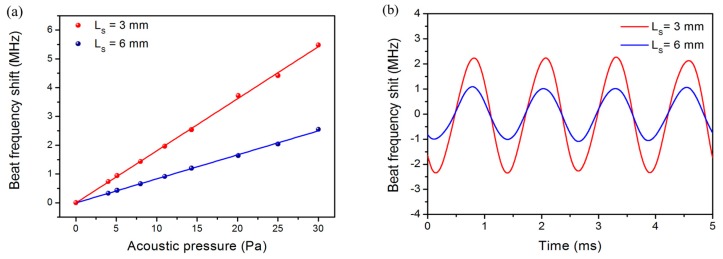
(**a**) Measured beat-frequency variations as a function of applied acoustic pressure at 800 Hz. *L*_s_ = 3 and 6 mm, respectively; (**b**) Output waveforms of the fiber laser hydrophones at 800 Hz, 25 Pa.

Figure 5a shows measured acoustically induced beat-frequency variations as a function of applied acoustic pressure. The result of two fiber laser hydrophones with *L_s_* = 3 and 6 mm are exhibited. The applied acoustic wave has a frequency of 800 Hz. The amplitudes vary in proportion with applied acoustic pressure. The measured sensitivities are 181 and 83 kHz/Pa, respectively, estimated from the linear fits. Figure 5b shows the output sinusoidal waveforms for an acoustic signal of 800 Hz, 25 Pa. 

**Figure 6:**
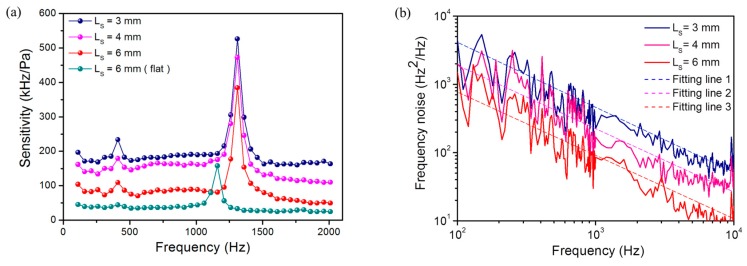
(**a**) Measured frequency responses of fiber laser hydrophones with different *L_s_*. In addition, measured result with a flat diaphragm as transducer is plotted for comparison; (**b**) Measured frequency noise spectra of fiber lasers with *L_s_* = 3, 4, and 6 mm.

We have tested the frequency responses of the fiber laser hydrophones with *L_s_* = 3, 4 and 6 mm, respectively. As shown in Figure 6a, the average sensitivities over the working bandwidth from 110 to 1210 Hz are 185.7, 159.6, and 85.9 kHz/Pa, respectively. According to Equation (4), the beat frequency change induced by a point load is in proportion to local intensity of the laser mode. As a result, shorter laser cavities enable higher local intensity and therefore higher sensitivities, because the laser modes are more confined. The natural resonant frequency of the corrugated diaphragm is 1310 Hz, determined by the elastic property and corrugation geometry. Figure 6a shows an abrupt change in sensitivity at about 400 Hz, which is probably a result of the mechanical resonance of the metal package. This weak resonance is not relevant with the mechanical property of the diaphragm and can be seen in each independent test. The measured acoustic sensitivity as well as the first-order resonant frequency are in good agreement with the calculated result. The slight difference between the calculated and measured sensitivities may be a result of the deviation from ideal corrugation geometry.

Figure 6a also shows the measured frequency response with a flat diaphragm for comparison. This diaphragm has the same diameter and thickness with the corrugated one. The natural resonant frequency of the flat diaphragm is about 1100 Hz. The average sensitivity is only 39.3 kHz/Pa with *L_s_* = 6 mm, much lower than the corrugated-diaphragm one. The comparison between the result obtained with a flat and a corrugated diaphragm shows the effect of the amplification factor *Γ,* which is determined by the diaphragm geometry. Specifically, a customized flat diaphragm with *H/h* = 0, *q* = 1 enables an amplitude *Γ* = 0.25. In contrast, the corrugated one with *H/h* = 10, *q* = 12 has an amplification factor *Γ* = 0.435, which is 74% higher than the flat one, in accordance with the measured result. In addition, a higher *H/h* ratio can yield an increased natural resonant frequency *f*_00_, based on the definition of Equations (A3) and (A5), which means the corrugated diaphragm can also provide a wider working bandwidth than a flat one.

The noise of the present hydrophone at the frequency range of interest mainly comes from the frequency noise of the fiber laser. The frequency noise mainly comes from the random thermal fluctuation of Er-doped fiber [22]. The spectral density has a 1/*f* dependence on frequency *f* and the spectrum is almost independent of pump power [23]. The beat signal has the same frequency-dependent profile but reduced strength, as a result of partial compensation between the two orthogonal lasing modes which are generated from the same cavity. Figure 6b shows the measured noise spectra of the beat signals with different cavity lengths. Here we simply write the spectrum as S(f)=C/f, where *C* is obtained by fitting lines in Figure 6b is used to characterize the noise level. The noise levels are *C* = 2 × 10^5^, 1.6 × 10^5^ and 1 × 10^5^ Hz^2^ for *L_s_* = 3, 4, and 6 mm, respectively. There is a peak at around 10^4^ Hz in the spectra, as a result of relaxation oscillation. The corresponding minimum detectable acoustic pressure *P_min_* can be simply estimated with $P_{min}=\sqrt{S\left(f\right)}/S_{b}$, where *S_b_* is the beat-frequency sensitivity. As a result, the minimum detectable acoustic pressures are estimated as 74, 78, and 112 µPa/Hz^1/2^ at 1 kHz, respectively, which are comparable to the zeroth order sea noise. With *L_s_* = 3 mm, the minimum detectable acoustic pressure reaches 74 µPa/Hz^1/2^ at 1 kHz, which is the best achieved result so far. In the experiment, the maximum applied pressure is 30 Pa in amplitude. The dynamic range of the hydrophone is around 112 dB.

**Figure 7:**
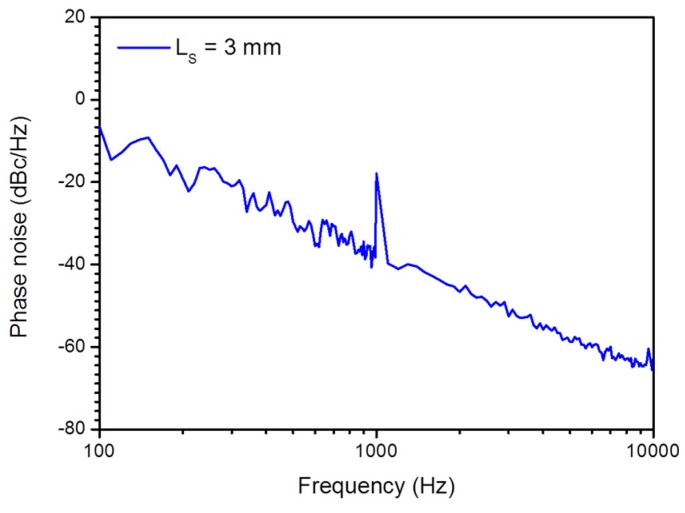
Recorded spectrum when applying 10 mPa acoustic signal at 1 kHz via phase noise measurement for *L_s_* = 3 mm.

The detection limit is also estimated via measuring a weak signal with an electronic spectrum analyzer. As shown in Figure 7, when applying 10 mPa acoustic pressure at 1 kHz with *L_s_* = 3 mm, the peak intensity of the beat signal at 1 kHz is about −18 dBc/Hz^1/2^ and the phase noise level is −39 dBc/Hz^1/2^. Considering that the measurement bandwidth of 100 Hz can cause a 20 dB intensity reduction for a single-frequency signal, the true SNR is 41 dB at 1 kHz. Therefore, the SNR is 21 dB and the NEAP is estimated as 83 µPa/Hz^1/2^ at 1 kHz, which basically agrees with the minimum detectable acoustic pressure 74 µPa/Hz^1/2^ at 1 kHz.

## 5. Conclusions

In summary, we have demonstrated a high-resolution beat-frequency-encoded fiber laser hydrophone based on an elastic corrugated diaphragm. The corrugated diaphragms are more pressure-sensitive than the flat ones, and therefore selected as a transducer to concentrate applied acoustic pressure into a near point load on the laser cavity. A higher corrugation depth-thickness ratio *H/h* enables a higher transduction efficiency. The concentrated load creates a local optical phase change and induces an optical response in beat-frequency shift. Experimental results show that shorter cavity length yields a higher sensitivity as well as a higher signal-to-noise ratio, as a result of more confined laser mode. As a result, the beat-frequency sensitivity reaches 185.7 kHz/Pa by use of a fiber laser with a grating separation length of 3 mm. The resolution of the hydrophone reaches 74 µPa/Hz^1/2^ at 1 kHz. The dynamic range of the hydrophone is estimated to 112 dB. The performance of the fiber laser hydrophone can be further improved forming lasers with highly confined mode, which requires rare-earth doped fibers with higher optical gain.

## Figures and Tables

**Figure A1:**
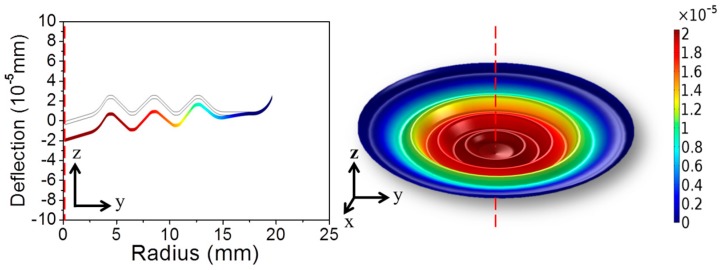
Calculated deformation of center-free corrugated diaphragm in response to 10 Pa static pressure.

## Appendix A. Theory of a Center-Free Corrugated Diaphragm

A pressure difference *P* can deform an edge-clamped diaphragm. The deformation can be simply depicted by the deflection *d_0_* at the rigid center, which can be expressed by [19]

(A1) $$\frac{Pr^{4}}{Eh^{4}}=A\cdot \frac{d_{0}}{h}+B\cdot \frac{{d_{0}}^{3}}{h^{3}}$$

where *E* and *µ* represent Young’s modulus and Poisson’s ratio of the metal, respectively, $A=2\left(3+q\right)\cdot \left(1+q\right)/3\left(1-\mu^{2}/q^{2}\right)$, $B=32\left(q+6-\mu \right)/\left[{\left(q+3\right)}^{2}\cdot \left(q-\mu \right)\right]$, and *q* is a shape factor defined as $q={\left(1+1.5H^{2}/h^{2}\right)}^{1/2}$ [16]. With the assumption of small deflection, the second term on the right side can be ignored, and Equation (1) can be simplified as

(A2) $$d_{0}=P\frac{r^{4}}{AEh^{3}}$$

Due to the similarity in *d_0_-P* dependence between Equation (2) and that of a flat diaphragm $d=Pr^{4}/64D$, where $D=Eh^{3}/12\left(1-\mu^{2}\right)$ denotes the flexural rigidity, the deformation of the corrugated diaphragm can be equivalently considered as a flat one with an effective flexural rigidity, which can be expressed by

(A3) $$D_{eff}=\frac{AEh^{3}}{64}$$

A diaphragm under harmonic perturbation can be approximately treated as a damping oscillator, and the induced harmonic deformation (in amplitude) can be simply described by [24]

(A4) $$d_{0}\left(\omega_{ƒ}\right)=d_{0}\frac{{\omega_{mn}}^{2}}{\sqrt{{\left({\omega_{mn}}^{2}-\omega_{ƒ}^{2}\right)}^{2}+4\omega_{ƒ}^{2}{\omega_{mn}}^{2}\xi^{2}}}$$

where *ξ* is the damping factor, *ω_f_* is the angular frequency of the acoustic wave, and *ω_mn_* is natural angular frequency which can be written as

(A5) $$\omega_{mn}=\frac{{\varphi_{mn}}^{2}}{r^{2}}\cdot \sqrt{\frac{D_{eff}}{\rho h}}$$

where $\varphi_{mn}$ is a constant related to the vibration mode of the diaphragm. For the fundamental mechanical mode, ${\varphi_{00}}^{2}=10.21$. Equation (A4) enables a flat response at below the lowest order natural resonant frequency *f*_00_. Therefore, we can treat the harmonic deformation at frequencies lower than the resonant frequency as a hydrostatic problem. The diaphragm deformation, as well as the sensor response, is calculated with this hydrostatic approximation in the text.

We have numerically calculated the deformation induced by a static pressure difference of 10 Pa based on finite element method. Figure A1 shows the calculated deformation of a center-free corrugated diaphragm. Its geometry is in accordance with the one in our experiment. The diaphragm presents maximum deflection at the rigid center.
